# Cerebrospinal fluid neutral lipids predict progression from mild cognitive impairment to Alzheimer’s disease

**DOI:** 10.1007/s11357-023-00989-x

**Published:** 2023-11-24

**Authors:** Farida Dakterzada, Mariona Jové, Raquel Huerto, Anna Carnes, Joaquim Sol, Reinald Pamplona, Gerard Piñol-Ripoll

**Affiliations:** 1 Unitat Trastorns Cognitius, Cognition and Behaviour Study Group, Hospital Universitari Santa Maria, IRBLleida, Rovira Roure No 44. 25198, Lleida, Spain; 2 Department of Experimental Medicine, University of Lleida, IRBLleida, Lleida, Spain; 3 Institut Català de La Salut, Lleida, Spain; 4 Research Support Unit Lleida, Fundació Institut Universitari Per a La Recerca a L’Atenció Primària de Salut Jordi Gol I Gurina (IDIAPJGol), Lleida, Spain

**Keywords:** Alzheimer’s disease, Mild cognitive impairment, Progression, Lipidomics, Cerebrospinal fluid, Neutral lipids

## Abstract

Genetic, metabolic, and clinical evidence links lipid dysregulation to an increased risk of Alzheimer’s disease (AD). However, the role of lipids in the pathophysiological processes of AD and its clinical progression is unclear. We investigated the association between cerebrospinal fluid (CSF) lipidome and the pathological hallmarks of AD, progression from mild cognitive impairment (MCI) to AD, and the rate of cognitive decline in MCI patients. The CSF lipidome was analyzed by liquid chromatography coupled to mass spectrometry in an LC-ESI-QTOF-MS/MS platform for 209 participants: 91 AD, 92 MCI, and 26 control participants. The MCI patients were followed up for a median of 58 (± 12.5) months to evaluate their clinical progression to AD. Forty-eight (52.2%) MCI patients progressed to AD during follow-up. We found that higher CSF levels of hexacosanoic acid and ceramide Cer(d38:4) were associated with an increased risk of amyloid beta 42 (Aβ42) positivity in CSF, while levels of phosphatidylethanolamine PE(40:0) were associated with a reduced risk. Higher CSF levels of sphingomyelin SM(30:1) were positively associated with pathological levels of phosphorylated tau in CSF. Cholesteryl ester CE(11D3:1) and an unknown lipid were recognized as the most associated lipid species with MCI to AD progression. Furthermore, TG(O-52:2) was identified as the lipid most strongly associated with the rate of progression. Our results indicate the involvement of membrane and intracellular neutral lipids in the pathophysiological processes of AD and the progression from MCI to AD dementia. Therefore, CSF neutral lipids can be used as potential prognostic markers for AD.

**Supplementary Information:**

The online version contains supplementary material available at 10.1007/s11357-023-00989-x.

## Introduction

Alzheimer’s disease (AD) is a human progressive neurodegenerative disease that results from age-related pathological processes. The exact etiology of AD is still unknown, and it is believed that apart from amyloid and tau pathologies, several other factors, including genetic, metabolic, bioenergetics, and environmental factors, also have a role in the onset and development of this disease [1, 2]. AD accounts for 60–70% of all dementia cases. The global prevalence of dementia was estimated at 57.4 million cases in 2019. This number is predicted to increase to 152.8 million cases in 2050 [3].

The disease is typically characterized by progressive memory impairment and subsequently by the gradual affectation of other mental abilities, such as behavior, speech, visuospatial orientation, and the motor system. The increasing decline in these cognitive capacities affects a person’s ability to perform daily routine tasks and finally leads to a complete loss of independence, disability, and death [4].

From a pathological point of view, AD is currently characterized by the accumulation of extracellular abnormally folded amyloid-beta (Aβ) protein into amyloid plaques, intracellular aggregations of hyperphosphorylated tau protein known as neurofibrillary tangles (NFTs), and synaptic and neuronal loss in the brain [5]. Clinically, the AD continuum can be divided into three stages: preclinical, mild cognitive impairment (MCI), and AD dementia [6]. Although both pathological and clinical manifestations of AD have a progressive nature, there is a weak synchronization between them. Amyloid deposition reaches its peak in the AD brain years before the beginning of clinical symptoms. Regarding tau pathology and synaptic loss, although they continue to progress in the symptomatic phase of AD, they also start many years before clinical manifestations become evident [5]. However, among these mechanisms, the loss of synapses and neuronal death have shown a stronger correlation with the clinical progression of the disease [7, 8].

Despite the new advances in the quantification and characterization of synaptic markers, the lack of specificity of these markers toward synaptic failure due to AD is their most important drawback [9]. This may mainly originate from our incomplete knowledge about the pathophysiological processes underlying synaptic deficits and subsequent neurodegeneration. The other issue is that there is high variability regarding the rate of MCI to AD progression among patients, with some having a faster course than others. To date, research has mainly focused on discovering the risk factors for the disease and the probability of developing AD regarding certain risk factors. Consequently, our knowledge about the factors that may affect the disease trajectory is highly limited [10].

The human brain is the most lipid-rich organ after adipose tissue and contains an incredible mixture of lipids [11]. Lipids are an important class of biomolecules that are involved in many vital cellular processes, including their role as building blocks of the cell membrane, cell signaling, and energy storage [12]. Several case‒control studies have associated dysregulation in various classes of lipids with AD development [13]. In addition, some clinical conditions highly related to lipid dysregulation, such as cardiovascular diseases, diabetes, and obesity, are among the most frequent comorbidities of AD [14, 15]. Apart from this clinical evidence, genetic studies have also revealed that genes involved in lipid metabolism are among the genes most associated with the risk of AD [16, 17]. Consistent with this, the inheritance of the apolipoprotein E epsilon 4 allele (*APOE ɛ4*) is the strongest genetic risk factor for AD. APOE is involved in the transport and metabolism of cholesterol [18]. Despite these strong links, little is known about the association between brain lipid alterations and pathological hallmarks of the disease. In addition, longitudinal studies investigating the association of lipids with AD progression and the rate of progression are lacking.

Biofluid analysis is the most convenient way to identify and monitor lipid dysregulations in patients. Blood, in particular, has been a preferable source because its acquisition is less invasive. As a result, the vast majority of studies have searched for AD-related lipid dysregulations in blood (plasma, serum, and blood cells) [19–22]. However, we should take into consideration that AD, although associated with many systemic abnormalities [14, 15], principally affects brain functionality. Therefore, cerebrospinal fluid (CSF) may be a more reliable and specific source for the examination of lipid alterations in AD because of its proximity and direct contact with brain tissue.

CSF is the biofluid with the closest relation to the brain, containing molecules of neural cell origin, which reflect, at least in part, brain metabolic activity. Among the CSF molecular profile, lipids are a preponderant component. It is estimated that the total lipid content of human CSF is around 0.2% of plasma levels [23], showing its own lipid profile in comparison to plasma (24). More specifically, lipidomic studies have estimated the presence of about 200–300 different lipid species, with a preferential quantitative presence of glycerophospholipids, followed by neutral lipids and sphingolipids [23, 24], and highlighting the triacylglycerides as the lipid class with the highest number of lipid species [25]. Consequently, CSF provides a valuable tool for exploring lipid homeostasis in patients with AD and identifying novel biomarkers for their diagnosis and prognosis.

In this context, the objectives of the present study were as follows: first, to determine the association between CSF lipidome and the clinical diagnoses of MCI and AD; second, to investigate the relationship between CSF lipidome and pathological hallmarks of the disease; third, to assess whether CSF lipids could be related to MCI to AD progression; fourth, to identify the lipids that could be associated with the rate of progression in MCI patients; and fifth, to evaluate whether changes in CSF lipid species could serve as prognostic biomarkers of progression and the rate of progression from MCI to AD.

## Methods

### Study population

The study participants were recruited consecutively from a sample of outpatients who visited the Cognitive Disorders Unit of the Hospital Universitari Santa Maria de Lleida from June, 2014, to December, 2016. The inclusion criteria for patients were as follows: (1) males and females without specific treatment for dementia at the moment of the inclusion, with a new diagnosis of MCI or mild and moderate AD (Mini-Mental State Examination (MMSE) ≥ 20). The diagnoses of MCI and AD were made according to the criteria of the National Institute on Ageing-Alzheimer’s Association [4, 26]; (2) absence of visual or hearing problems that, in the investigator’s judgement, would decrease the compliance with the neuropsychological examination; (3) an informed consent form signed by the patient and the responsible caregiver (and/or if applicable, the legal representative if different from the responsible caregiver); and (4) a knowledgeable and reliable caregiver who accompanied the patient to all clinic visits during the study.

Controls were subjects without neurological or neuropsychiatric diseases who underwent lumbar puncture for any other reason. Epidemiological data, including age, sex, education, and the time of symptom onset, were recorded using a structured interview conducted during the initial patient visit. In addition, blood analytical data, including complete blood count (CBC) and lipid profile were also registered for each subject.

For AD and MCI patients, the exclusion criteria were as follows: (1) a diagnosis of dementia other than AD or any somatic, psychiatric, or neurological disorder that might cause cognitive impairment; (2) mild-to-moderate AD with current acetylcholinesterase inhibitor treatment or memantine; (3) presence of serious comorbidities: cancer, excessive intake of alcohol (> 280 g/week), severe depression, severe renal or hepatic insufficiency, severe cardiac or respiratory failure; (4) investigational drug or device use; (5) patient or family declining to take part; (6) CT scan or MRI evidence of hydrocephalus, stroke, a space-occupying lesion, cerebral infection, or any clinically significant central nervous system disease other than AD; (7) mental retardation, organic mental disorders, or mental disorders due to a general medical condition (DSM-IV-TR™ criteria [27]); (8) suffering from thyroid and/or vitamin B12 deficiency. Patients with vitamin B12 or folate deficiency could be enrolled in the study provided they had been on a supplement therapy for > 3 months prior to the screening visit, and the levels of vitamin B12 or folate were stable. Patients with thyroid disease could be enrolled in the study provided they were stable and euthyroid.

The cognitive state of the study population was assessed using MMSE [28] at baseline. The MCI patients were followed up for a median of 58 (± 12.5) months to assess their progression to AD. For these patients, the MMSE was administered at each annual visit until the end of the follow-up. The final score of MMSE was adjusted by age and educational level. Progressive cognitive deterioration from MCI to AD was defined as (1) losing more than 3 points between the first and last MMSE, (2) having dementia at follow-up, or (3) scoring less than 24 on the last MMSE [29]. Therefore, based on the follow-up data, we divided the MCI group into progressive and nonprogressive MCI patients to assess the association of CSF lipids with MCI to AD progression. The rate of progression from MCI to AD dementia was defined as the time between the baseline visit and the date of the diagnosis of AD. The study was approved by the local ethics committee (CEIm 1374).

### Sample collection

Fasting blood and CSF samples were collected between 8:00 and 10:00 a.m. CSF samples were collected by a lumbar puncture in polypropylene tubes and subsequently centrifuged at 2000 × g for 10 min at 4 °C to exclude cells or other insoluble material. Blood samples were collected in EDTA-containing tubes and centrifuged at 1500 rpm for 20 min to obtain plasma and the buffy coat. The buffy coat was used for DNA extraction and subsequent *APOE* genotyping. All samples were aliquoted and immediately stored at − 80 °C until use. Samples were obtained with support from IRBLleida Biobank (B.0000682) and PLATAFORMA BIOBANCOS PT17/0015/0027, following the guidelines of Spanish legislation on this matter (Real Decreto 1716/2011).

### AD biomarker measurement

The levels of CSF amyloid beta 1–42 (Aβ42) (INNOTEST® β-AMYLOID (1–42)), total tau (Ttau) (INNOTEST® hTAU Ag), and phosphorylated tau (Ptau) (INNOTEST® PHOSPHO-TAU (181P)) were determined by ELISA based on the manufacturer’s instructions (Fujirebio Europe, Ghent, Belgium). We used our own cut-off points that were previously calculated based on another study population. Thus, we considered Aβ42 values < 600 pg/mL, Ttau > 425 pg/mL, and Ptau > 65 pg/mL as positive/abnormal [30].

### APOE genotyping

DNA was extracted automatically from the buffy coat cells using the Maxwell RSC Buffy Coat DNA Kit (Promega Biotech Ibérica SL, Madrid, Spain) and the Maxwell RSC instrument according to the manufacturer’s instructions. Two microliters of extracted DNA was used for *APOE* genotyping by real-time PCR according to the TaqMan® SNP genotyping assay user guide (Publication Number MAN0009593, revision B.0).

### Lipidomics

The CSF lipidome was analyzed using an untargeted lipidomic approach. The lipids were extracted using a methanol tert butyl ether-based validated method [31, 32]. Class representative internal standards (Supplementary Table 1) were added to the extraction solvent to check lipid species retention time, to evaluate lipid extraction for each sample, and to use as an internal standard for the semiquantitative approach used. Lipid extracts were analyzed by liquid chromatography‒mass spectrometry (LC‒MS) using an Agilent UPLC 1290 liquid chromatograph coupled to an Agilent Q-TOF MS/MS 6520 mass spectrometer (Agilent Technologies, Barcelona, Spain) as previously described [33, 34]. The samples were injected in a random order, and quality control (QC) samples were distributed at five-sample intervals to control instrumental drift. QC samples were pools of all the samples distributed in different aliquots. Data were acquired in both positive and negative ionization modes. For MS/MS confirmation, the same parameters used for MS analyses were applied, adding collision voltages of 0 V, 10 V, 20 V, and 40 V. Data were acquired using MassHunter Data Acquisition software (Agilent Technologies) and preprocessed using MassHunter Mass Profiler Professional software (Agilent Technologies), as previously described [34]. Compounds from different samples were aligned using retention time windows of 0.1% ± 0.25 min and 30 ppm ± 2 mDa. Only features with a minimum of 2 ions and stable features (found in at least 70% of the QC samples) were taken into consideration for the analysis and correction of individual bias [35]. The signal was corrected using a LOESS approach [36].

### Lipid identification

The potential identity of the differentially expressed features, defined by exact mass and retention time, was searched in the Human Metabolome Database (HMDB) [37], while the molecular weight tolerance was adjusted to 30 ppm. The adducts considered for the HMDB search were the following: positive ionization: M + H, M + NH4, M + NH4-H2O, M + Na, M + K, and M + 2 K-H; negative ionization: M-H, M-H2O-H, M + C2H3O2, and M + HCO2. Potential identities were confirmed through the comparison of the exact mass and MS/MS spectra fragmentation pattern of the class representative internal standards, when available, with the public database [34], as well as through the comparison of the retention time with the expected retention times of the chromatographic methodology used (lysophospholipids: 0–3 min; fatty acyls: 0–3.5 min; phospholipids, sphyngomyelins, and diacylglycerides: 3–7 min; and triacylglycerides and cholesteryl esters: 7.5–10.5 min) [33] and with the retention times of class-representative internal standards.

### Statistical analysis

One-way ANOVA (or nonparametric Kruskal‒Wallis) and chi-square (or Fisher’s exact) tests were used for the analysis of the quantitative and qualitative variables among the three diagnostic groups, respectively. Student’s *t* (or the Mann‒Whitney *U*) and chi-square (or Fisher’s exact) tests were used for the analysis of quantitative and qualitative variables between the progressive and nonprogressive MCI groups. The quantitative variables are presented as the means (± standard deviation, SD) or medians (25th; 75th percentiles), and the qualitative variables are presented as percentages (frequency).

The cross-sectional association of experimental variables with quantitative outcomes (the levels of Aβ42, Ttau, and Ptau in CSF and the rate of progression) was assessed using Spearman’s correlation. The cross-sectional association of the experimental variables with categorical outcomes (diagnosis, Aβ42 status, Ttau status, Ptau status, and progression/no progression) was studied using logistic regression analysis. Cox hazard analysis was used to assess the association of variables with the rate of progression. For regression analyses, the values corresponding to each independent variable were dichotomized by their median, and the high value (> median) of each variable was compared to its low value (≤ median). In addition, to determine whether the logistic regression models fit our data well, a Hosmer‒Lemeshow test was performed for each model. The Hosmer‒Lemeshow statistic indicates a poor fit if its significance value is less than 0.05. When analyzing variables associated with progression, the AUC of the regression model, including lipids, was compared to the same model without lipids with the Hanley-McNeil test [38]. Values of *z* above the cut-off were taken as evidence that the “true” ROC areas were different. To minimize the negative effects of overfitting, step-by-step forward selection with conditional criteria was applied as a criterion for the input of the variables into the regression models. The selection of variables by steps also allows the detection of multicollinearity. Although the detection of multicollinearity increases the precision of estimated coefficients and the power of the statistical analysis, some variables that may be highly related to the dependent variables will be lost (e.g., features from the same metabolic pathway). To overcome this problem, after running each regression analysis, we eliminated the variables that had been inputted into the model, and the analysis was run again to let other influential lipids, if they existed, enter the model. This process was continued until the AUC of the regression model reached < 80. Finally, all of the statistical analyses were adjusted for age, sex, *APOE ɛ4* allele status, MMSE score, and, if applicable, appropriate AD CSF biomarkers (Aβ42, Ttau, and Ptau), including these parameters as predictors. All statistical analyses were performed using IBM SPSS version 25 (SPSS Inc., Chicago, IL, USA).

## Results

### Study population

Our study population included 209 participants who were divided into three diagnostic groups: 91 (43.5%) AD, 92 (44.6%) MCI, and 26 (12.4%) control (CTL) participants (Table 1). Our results showed that 44 patients (47.8%) remained cognitively stable, while 48 patients (52.2%) progressed to AD dementia after a median follow-up of 58 (± 12.5) months (Table 2). None of the MCI patients progressed to non-AD dementia.

**Table 1:** Characteristics of the study population based on the differential diagnosis

	Total (*N* = 209)	AD (*N* = 91)	MCI (*N* = 92)	CTL (*N* = 26)	*p*
Demographic data					
Age	74 [70;78]	76 [72;80]	73 [69; 77]	66 [60;74]	< 0.001
Sex (female)	54.3% (114)	59.3% (54)	51.1% (47)	50% (13)	0.437
Education					0.074
Illiterate	8% (14)	7.2% (6)	6.5% (6)	20.0% (2)	
Primary	68.5% (120)	78.0% (65)	59.0% (49)	60.0% (6)	
Secondary	17.7% (31)	12.0% (10)	23.1% (19)	20.0% (2)	
University	5.7% (10)	2.4% (2)	9.7% (8)	0% (0)	
Comorbidities					
Depression	33.9% (71)	30.7% (28)	42.3% (39)	15.3% (4)	0.029
Hypertension	56.4% (118)	57.1% (52)	60.8% (56)	38.4% (10)	0.138
Stroke	3.8% (8)	5.4% (5)	2.1% (2)	3.8% (1)	0.496
Diabetes mellitus	20.5% (43)	19.7% (18)	21.7% (20)	19.2% (5)	0.945
Dyslipidemia	40.1% (84)	47.2% (43)	33.6% (31)	38.4% (10)	0.154
Complete blood count					
Hemoglobin (g/dL)	13.7 (1.72)	13.9 (1.46)	13.5 (1.62)	13.1 (3.45)	0.158
Hematocrit (%)	41.5 (5.07)	42.4 (4.35)	41.0 (5.02)	40 (9.15)	0.093
WBC (× 10^9^/L)	7.1 [5.9;8.5]	6.9 [5.8;7.9]	7.6 [6.3;10.0]	6.7 [5.7;10.4]	0.073
Platelet (× 10^9^/L)	224 [195;264]	222 [195;254]	216 [187;282]	265 [218;282]	0.399
CSF AD biomarkers					
Aβ42 (pg/mL)	551 [420;729]	493 [395;583]	595 [435;864]	1029 [634;1331]	< 0.001
Ttau (pg/mL)	400 [248;601]	494 [357;705]	334 [229;542]	247 [139;313]	< 0.001
Ptau (pg/mL)	67.35 [48;92]	81 [54; 98]	63 [44;87]	45 [30;63]	< 0.001
MMSE score	25 [23;28]	23 [22;25]	27 [25;28]	30 [28;30]	< 0.001
*APOE ɛ4*	43.4% (86)	51.6% (47)	40.2% (37)	7.7% (2)	0.003

*AD* Alzheimer’s disease, *MCI* mild cognitive impairment, *CTL* control, *MMSE* Mini-Mental State Examination, *Aβ42* amyloid beta 1–42, *Ttau* total tau, *Ptau* phosphorylated tau, *APOE ɛ4* apolipoprotein E ɛ4 allele, *P* values were calculated by comparing diagnostic groups using one-way ANOVA (or nonparametric Kruskal‒Wallis test) for quantitative variables and chi-square test for qualitative variables

**Table 2:** Characteristics of the progressive and nonprogressive MCI patients

	Total MCI (*N* = 92)	Progressive (*N* = 48)	Nonprogressive (*N* = 44)	*p*
Demographic data				
Age	72 (6.0)	73 (6.0)	72 (5.4)	0.328
Sex (female)	50% (46)	52.1% (25)	47.7% (21)	0.673
Education				0.549
Illiterate	7.3% (6)	10.6% (5)	2.9% (1)	
Primary	59.8% (49)	55.3% (26)	65.7% (23)	
Secondary	23.2% (19)	23.4% (11)	22.9% (8)	
University	9.8% (8)	10.6% (5)	8.6% (3)	
Comorbidities				
Depression	42.3% (39)	41.6% (20)	43.1% (19)	0.817
Hypertension	60.8% (56)	54.1% (26)	68.1% (30)	0.137
Stroke	2.1% (2)	0% (0)	4.5% (2)	0.131
Diabetes mellitus	21.7% (20)	18.7% (9)	25% (11)	0.437
Dyslipidemia	33.6% (31)	29.1% (14)	38.6% (17)	0.304
Complete blood count				
Hemoglobin (g/dL)	13.5 (1.62)	13.1 (1.34)	13.7 (1.77)	0.255
Hematocrit (%)	41.0 (5.02)	40.3 (4.24)	41.5 (5.50)	0.221
WBC (× 10^9^/L)	7.6 [6.3;10.0]	6.7 [5.6;8.4]	8.2 [6.4;10.7]	0.061
Platelet (× 10^9^/L)	216 [187;282]	209 [159;232]	235 [196;286]	0.241
CSF AD biomarkers				
Aβ42 (pg/mL)	589[432;864]	478 [374;619]	798 [582;928]	< 0.001
Ttau (pg/mL)	333 [227;534]	447 [259;709]	265 [198;353]	< 0.001
Ptau (pg/mL)	64 [43;86]	76 [49;107]	54 [40;66]	0.001
MMSE score	27 [25;28]	26 [24;28]	27 [26;29]	0.063
*APOE ɛ4*	40.2% (37)	63% (29)	19.5% (8)	< 0.001

*MCI* mild cognitive impairment, *WBC* white blood cell, *CSF* cerebrospinal fluid, *Aβ42* amyloid beta 1–42, *Ttau* total tau, *Ptau* phosphorylated tau, *MMSE* Mini-Mental State Examination, *APOE ɛ4* apolipoprotein E ɛ4 allele. *P* values were calculated by comparing groups using Student’s *t*-test (or Mann–Whitney U test) for quantitative variables and Pearson’s chi-square test for qualitative variables

### CSF lipids associated with the diagnoses of MCI and AD

The CSF samples were analyzed in positive and negative ionization modes. After baseline correction, peak picking and alignment, and further corrections, including quality control assessment, filtering, and the correction of the signal, 201 features remained for evaluation, among which 174 features were detected in positive and 27 in negative ionization mode. Our analysis detected no lipids associated with the diagnosis of MCI or AD versus the control. In addition, after analyzing the whole CSF lipidome, we did not identify any lipid profile specific to any individual diagnostic group (Supplementary Figs. 1 and 2).

### CSF lipids associated with CSF measures of AD pathology

The association of lipid species with amyloid pathology was evaluated by comparing the CSF lipidome profile between participants with abnormal levels of Aβ42 in CSF and participants with normal levels of this biomarker. Our analysis detected hexacosanoic acid C26:0 (*p* < 0.001), ceramide Cer(d38:4) (*p* = 0.007), phosphatidylethanolamine PE(40:0) (*p* = 0.007), and two unknown lipids (mass 746.7401, RT 7.47 (*p* < 0.001) and mass 1464.461, RT 10.27 (*p* = 0.001)) as the most associated CSF lipids with Aβ42 positivity in CSF (Table 3). Regarding the association of CSF lipids with Ptau positivity in CSF, our analysis detected sphingomyelin SM(30:1) (*p* = 0.01) as the most associated lipid (Table 3), and an unknown lipid (mass 636.5484, RT 8.21, (*p* < 0.047)) was associated with the pathological levels of Ttau (Supplementary Table 2). Other lipids significantly associated with each AD-related pathology are presented in Supplementary Table 2.

**Table 3:** CSF lipids associated with the positivity of each AD-related CSF biomarker

	Lipid name	Mass	RT	*p*	OR	99% CI for OR
Aβ42	C26:0	396.3861	3.92	< 0.001	0.159	0.049–0.521
	Unknown	746.7401	7.47	< 0.001	6.729	1.973–22.956
	Cer(d38:4)	587.5138	7.85	0.007	0.302	0.096–0.946
	PE(40:0)	803.6014	8.03	0.007	3.696	1.063–12.850
	Unknown	1464.461	10.27	0.001	5.146	1.419–18.665
Ptau	SM(30:1)	692.5416	8.73	0.010	5.349	1.472–19.442

*RT* retention time, *OR* odds ratio, *CI* confidence interval, *Aβ42* amyloid beta 1–42, *Ptau* phosphorylated tau, *Ttau* total tau, *C26:0* hexacosanoic acid, *Cer* ceramide, *SM* sphingomyelin

### CSF lipids associated with progression and the rate of progression from MCI to AD

The association of lipids with MCI to AD progression was evaluated by comparing the CSF lipidome profile of patients who progressed to AD (*N* = 48) with that of patients who did not progress (*N* = 44) to AD during the follow-up. After adjusting for age, sex, *APOE ɛ4* status, MMSE score, and AD CSF biomarkers, higher CSF levels of cholesteryl ester CE(11D3:1) (*p* = 0.01) and an unknown lipid (mass 528.4519, RT 8.59) (*p* = 0.048) were associated with a significantly greater risk of MCI to AD progression (Table 4). A regression model consisting of these two lipids and covariates yielded an AUC = 0.88 (*p* < 0.001, 95% CI 0.79–0.97), and the Hosmer‒Lemeshow test yielded *p* = 0.357. The AUC of the statistical model without these significantly associated lipids was 0.86 (*p* < 0.001, 95% CI 0.762–0.962), which was not significantly different from the model including these lipids (*z* = 0.425, |*z*|< 1.96).

**Table 4:** CSF lipids associated with progression from MCI to AD

Lipid name	Mass	RT	*p*	OR	95% CI for OR
Unknown	528.4519	8.59	0.048	0.219	0.048–0.989
CE(11D3:1)	688.5836	9.17	0.010	9.288	1.670–51.647

*RT* retention time, *OR* odds ratio, *CI* confidence interval, *CE* cholesteryl ester

**Fig. 1:**
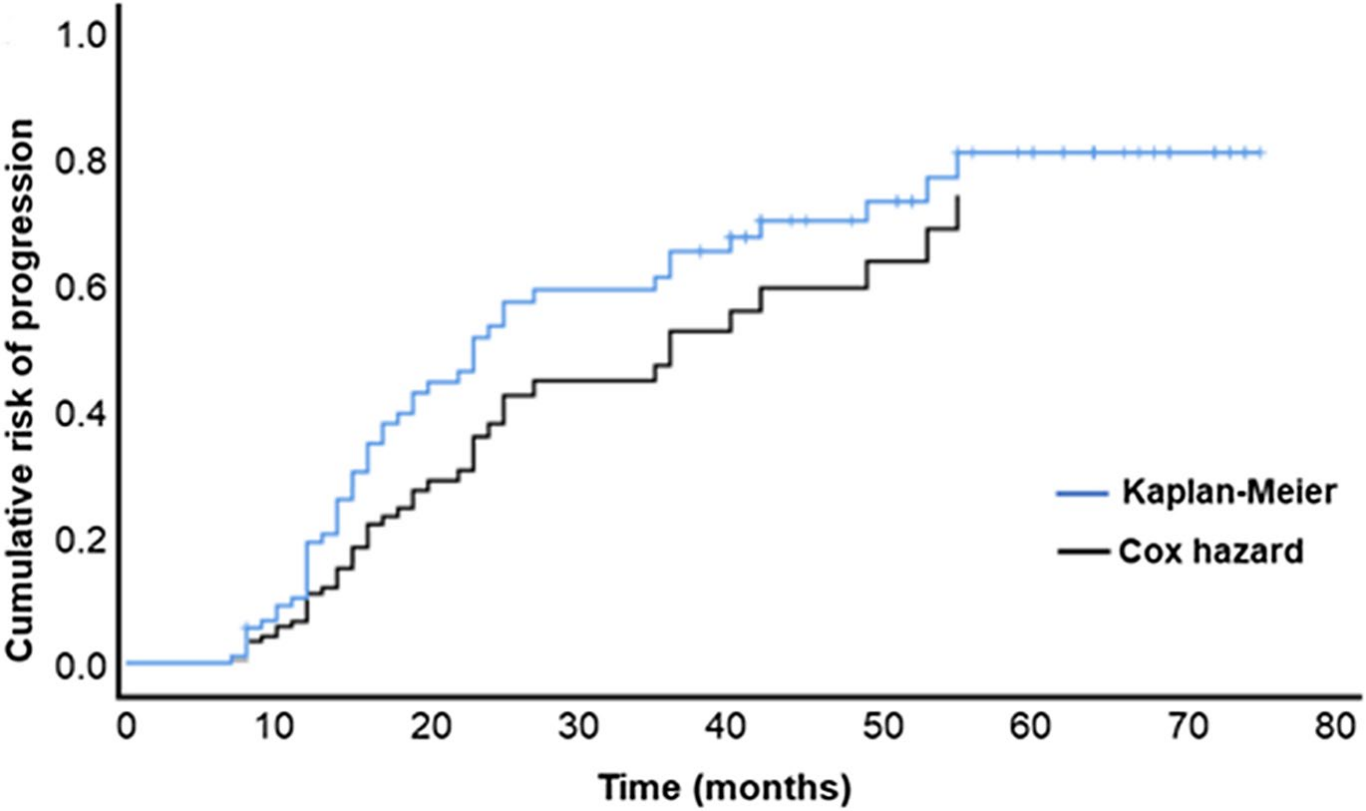
Prediction of the rate of MCI to AD progression. Rate of progression predicted by the Cox hazard model (black line) compared to the rate of progression based on the clinical data that were calculated by Kaplan‒Meier analysis (blue line)

The Cox hazard analysis detected ether-linked triglyceride TG(O-52:2) in CSF as the most influential lipid on the rate of progression. Higher levels of this lipid in CSF were associated with a higher rate of progression (*p* < 0.001) (Table 5). The comparison of the predictive performance of the Cox model including TG(O-52:2) as a predictor with the real rate of progression calculated by Kaplan‒Meier analysis showed that the model has good predictive power regarding the rate of MCI to AD progression (Fig. 1). Five other lipid species, including two phosphatidic acids and a ceramide, were also associated with the rate of MCI to AD progression (Supplementary Table 3).

**Table 5:** Plasma lipids associated with the rate of progression from MCI to AD

Name	Mass	RT	*p*	OR	99% CI for OR
Aβ42			< 0.001	0.997	0.995–0.999
Ttau			< 0.001	1.002	1.001–1.003
TG(O-52:2)	861.8141	10.4	0.003	2.700	1.138–6.403

*RT* retention time, *OR* odds ratio, *CI* confidence interval, *Aβ42* amyloid beta 1–42, *Ttau* total tau, *TG(O)* ether-linked triglyceride

## Discussion

We performed an untargeted lipidomic analysis aimed at identifying CSF lipids associated with the clinical diagnosis of AD and the levels of AD CSF biomarkers. We also searched for the association of CSF lipids with the progression and rate of progression from MCI to AD. The data were adjusted for age, sex, MMSE score, *APOE ɛ4* status, and levels of CSF AD biomarkers. When searching for the association of lipids with each CSF biomarker, we adjusted the data for two other CSF biomarkers to identify specific lipid alterations related to that biomarker. Controlling for the CSF AD biomarkers also permitted us to explore the involvement of lipids in disease progression independent of their possible role in the alterations of core AD biomarkers. Despite controlling for several variables, we identified sets of CSF lipids that were associated with each AD biomarker and MCI to AD progression, suggesting that the role of lipids in AD pathology and progression is broader than their possible role in the development of pathological hallmarks of AD and the influence of *APOE ɛ4*.

We found no association between detected CSF lipid species and the diagnoses of MCI and AD vs. control. This dissociation between the current parameters of both MCI and AD diagnoses and the CSF lipidome was also reported previously. In a study by Wood et al., these investigators found no lipid alteration in postmortem CSF of demented patients compared to controls except for decreased levels of docosahexaenoic acid in MCI and demented patients compared with control participants [39]. In another study, Toledo et al. found no association between the dysregulation of serum lipid species and the differential diagnosis of MCI and AD vs. control. In the latter study, the detection of lipid dysregulations was observed only after the substratification of the diagnostic groups (control, MCI, and AD) based on CSF biomarkers [21]. This lack of association between lipids and differential diagnosis of patients in the AD continuum that was observed in our study and some previous studies might be a consequence of a divergent mechanistic relationship but may also indicate the importance of the definition of diagnostic groups based on biological pathology. The other possibility that may have led to this lack of association might be the methodological constraints imposed in our analysis, which may have led to a lack of convergence in our statistical models.

Our analysis associated several lipid species with pathological levels of Aβ42 in CSF. The most associated lipids with a known identity were C26:0, Cer(d38:4), and PE(40:0). C26:0 is a saturated very long-chain fatty acid (VLCFA). In line with our finding, Iuliano et al. found lower levels of C26:0 in the plasma of AD and aMCI patients compared to controls [40]. However, in a study by Zarrouk et al., plasma and red blood cell levels of C26:0 were reported to be significantly higher in demented patients than in control participants [41]. Nevertheless, it has been shown that there is no correlation between most CSF and plasma lipids and, therefore, the comparison of lipids in these two biofluids may not be entirely correct and they may not be used interchangeably [24, 32].

An in vitro experiment showed that C26:0 increased amyloid precursor protein (APP) processing and Aβ42 generation [42]. However, there is no previous report concerning the effect of C26:0 on the production/clearance of Aβ42 in vivo. A brain tissue analysis showed higher levels of VLCFAs in Braak stage V–VI compared to stage I–II and higher levels of brain cortical C26:0 in stage V–VI compared to stage I–II and III–IV [43].

VLCFAs, including C26:0, are metabolized in peroxisomes. This result may indicate the involvement of peroxisomes in amyloid pathology. Although peroxisomal dysfunction has been previously reported in AD [43–45], a direct effect of peroxisomal dysfunction on amyloid pathology has not been studied. Some peroxisome proliferator-activated receptor-alpha (PPARα) ligands have been shown to reduce amyloid plaque pathology in transgenic animal models of AD [46]. Furthermore, the ADAM10 gene has been demonstrated to be a PPARα target [47]. Therefore, it is possible that PPARα mediates both APP processing and peroxisomal lipid homeostasis, and therefore, its dysregulation in AD [48, 49] affects both processes.

Ceramides and phospholipids are structural constituents of biological membranes where APP processing occurs. It is now well known that membrane composition can affect the activity of membrane-embedded enzymes, including those involved in APP processing [50–52]. In addition, they can have roles as bioactive molecules in a variety of biological events that can be involved in Aβ production, such as inflammation and oxidative stress [53]. In turn, Aβ can stimulate ceramide production by activating sphingomyelinase, which converts SM into ceramide [54, 55]. Furthermore, ether-linked phospholipids may protect other membrane lipids against oxidation [56].

We found that higher CSF levels of SM(30:1) were associated with Ptau positivity in our study population. In agreement with our results, Varma et al. reported a positive correlation between brain levels of several SM species and disease severity determined by Braak scores [20]. SM is highly enriched in myelin sheaths. Myelin sheaths produced by oligodendrocytes cover axonal projections, where large quantities of tau are localized. This proximity may also suggest some bidirectional impact between SMs and tau protein. In addition, in oligodendrocytes, the proteins, and messenger RNAs necessary for myelination should be translocated to their target site at the tips of very long processes via cytoskeleton translocation machinery [57]. The hyperphosphorylation of tau disrupts tau sorting into these projections and interferes with the sorting mechanism that underlies myelin formation [58]. A recent finding suggests that oligodendrocytes may have a role in the seeding and spreading of Ptau [59]. It has also been shown that some tau phosphorylation kinases affect myelination [60, 61]. Interestingly, recent evidence points to the possible role of sphingolipid biosynthesis in the phosphorylation of tau protein [62]. However, additional studies are required to understand the functional consequences of these dysregulations on AD pathology and vice versa.

Our analysis identified two lipid species associated with MCI to AD progression: a CE and an unknown lipid. We found that higher CSF levels of CE(11D3:1) were associated with an increased risk of progression. These two lipids slightly increased the predictive value of the statistical model from 0.86 to 0.88, indicating that lipids can have an additive value to the predictive power of known markers (markers of pathology, the presence of the *APOE ɛ4* allele, and baseline cognition) that affect the progression from MCI to AD.

The accumulation of CEs in lipid droplet (LD, the storage site for neutral lipids, including CE and TG) organelles has been reported in the AD brain [63, 64] and in AD transgenic mice [65–67]. We previously found that plasma neutral lipid dysregulations were associated with MCI to AD progression [68]. Some previous lipidomic studies have linked plasma and CSF levels of CE species with the diagnoses of MCI and AD [69]. CE species have also been shown to be modulators of amyloid [70, 71] and tau pathologies [72]. Therefore, by regulating amyloid and tau pathologies, intracellular levels of cholesterol, in the form of CEs, could play an important role in neurodegeneration. On the other hand, cholesterol, as a main component of cellular membranes, has been demonstrated to play fundamental roles in synaptic plasticity and function in the brain [73]. Therefore, dysregulation in brain cholesterol homeostasis would affect cognitive abilities, as evidenced recently [74]. In addition, LDs have been shown to be active signaling organelles that regulate processes such as proteasome activity, inflammation, and oxidative stress, all of which are possible drivers of neuronal injury and cell death [75].

The association we found between higher levels of CE in CSF and increased risk of progression may be the cause or consequence of neurodegeneration. If it is the cause, there may be some problem in the transport of cholesterol from astrocytes to neurons [76] or the transport of this molecule from the brain to the periphery [77, 78]. These processes have been associated with a more rapid course of cognitive decline in later life [79]. If it is the consequence of neurodegeneration, it may indicate that dying neurons generate high levels of cholesterol-rich debris that could be swallowed by glial cells and lead to increased intracellular cholesterol in the form of CE in these cells (80). Nevertheless, our data, for the first time, link higher levels of CEs in CSF with an increased risk of progression from MCI to AD.

Based on our findings, higher CSF levels of ether-linked triglyceride TG(O-52:2) were associated with a faster rate of progression. In an agreement with this result, our previous study also related plasma TG(O) dysregulation to the rate of MCI to AD progression [68]. TG(O) are lipid species that have been found in LDs. The exact role of TG(O) lipids in cell biology is not clear. One of the possible functions of TG(O) in LDs could be the protection of FAs attached to other TGs in LDs from oxidative stress. This function has been demonstrated for their phospholipid counterparts (either phospholipids or plasmalogens), whose presence in the membrane protects other lipids against oxidation (56). In the AD brain, it seems that the accumulation of LDs is more pronounced in glia. This increase has been related to increased oxidative stress in the AD brain and the role that glia have in the detoxification and storage of oxidized lipids [81]. Previous studies have found elevated lipoxidation markers in AD and MCI brains [82]. Therefore, the formation of LDs in AD could be a strategy for delaying neurotoxicity and neuronal death and, as a result, could affect time to progression. However, this strategy eventually fails because of the limited capacity of glia [83]. Whether neutral lipid dysregulation is the cause or consequence of neurodegeneration or both, our data link them to the cognitive impairment and the clinical progression of MCI patients. Therefore, the measurement of these lipids may have prognostic value in these patients.

Our study has some strengths and limitations. The strengths of our study include the following: first, we evaluated the association of lipids with each AD biomarker by controlling for other core AD biomarkers. This analysis permitted us to discover lipids that are specifically associated with each AD pathological hallmark. Second, our MCI group had a long follow-up period that increased the accuracy of our defined groups as progressive or nonprogressive MCI. Third, for the first time, we assessed the association of lipids with AD diagnosis and progression, independent of their possible role in known pathological hallmarks of the disease. However, we did not access data regarding medication and diet that may have affected our results and should be taken into consideration for future studies. Furthermore, there is a need for studies, especially at the tissue level, to connect metabolic changes within a pathway and network context.

In conclusion, our results indicated that CSF lipids were associated with CSF measures of AD pathology. With respect to the progression from MCI to AD dementia, our results suggest that neutral lipids are involved in the pathophysiological processes underlying neurodegeneration. In addition, dysregulated lipids in CSF may be useful biomarkers for the prediction of the progression and rate of progression from MCI to AD.

### Supplementary Information

Below is the link to the electronic supplementary material.

Supplementary file1 (DOCX 789 KB)

## Data Availability

The data reported in this manuscript are available within the article and/or its supplementary data. Additional data will be shared by request from any qualified investigator.
