# Supplementary material for: Cerebrospinal fluid neutral lipids predict progression from mild cognitive impairment to Alzheimer’s disease
Source: GeroScience. 2023 Nov 24;46(1):683–96. doi: 10.1007/s11357-023-00989-x (PMC10828158; doi:10.1007/s11357-023-00989-x)
Supplement: Supplementary file 1 — Supplementary file1 (DOCX 789 KB) [file 11357_2023_989_MOESM1_ESM.docx]

**Supplementary data**

**Cerebrospinal fluid neutral lipids predict progression from mild cognitive impairment to Alzheimer’s disease**

Farida Dakterzada^1^, Mariona Jové^2^, Raquel Huerto^1^, Anna Carnes^1^, Joaquim Sol ^2,3,4^, Reinald Pamplona^2^, Gerard Piñol-Ripoll^1^*

1. Unitat Trastorns Cognitius, Cognition and Behaviour Study Group, Hospital Universitari Santa Maria, IRBLleida, Lleida, Spain.

2. Department of Experimental Medicine, University of Lleida, IRBLleida, Lleida, Spain.

3. Institut Català de la Salut, Lleida, Spain.

4. Research Support Unit Lleida, Fundació Institut Universitari per a la Recerca a l'Atenció Primària de Salut Jordi Gol i Gurina (IDIAPJGol), Lleida, Spain.

* Corresponding author:

Gerard Piñol-Ripoll

Cognitive Disorders Unit

Hospital Universitari Santa Maria.

Rovira Roure n° 44. 25198. Lleida. Spain

Telephone: 34-937-727222. Ext. 173. Fax: 34-976-727366

E-mail: [gerard_437302@hotmail.com](mailto:gerard_437302@hotmail.com)

**Supplementary Table 1.** Class representative and extraction internal standards added to the samples.

| **Compound** | **Reference**  **(Catalogue number, provider)** |
| --- | --- |
| 1,3(d5)-dihexadecanoyl-glycerol | 110537, Avanti Polar Lipids |
| 1,3(d5)-dihexadecanoyl-2-octadecanoyl-glycerol | 110543, Avanti Polar Lipids |
| 1-hexadecanoyl(d31)-2-(9Z-octadecenoyl)-sn-glycero-3-phosphate | 110920, Avanti Polar Lipids |
| 1-hexadecanoyl(d31)-2-(9Z-octadecenoyl)-sn-glycero-3-phosphocholine | 110918, Avanti Polar Lipids |
| 1-hexadecanoyl(d31)-2-(9Z-octadecenoyl)-sn-glycero-3-phosphoethanolamine | 110921, Avanti Polar Lipids |
| 1-hexadecanoyl-2-(9Z-octadecenoyl)-sn-glycero-3-phospho-(1'-rac-glycerol-1',1',2',3',3'-d5) | 110899, Avanti Polar Lipids |
| 1-hexadecanoyl(d31)-2-(9Z-octadecenoyl)-sn-glycero-3-phospho-myo-inositol | 110923, Avanti Polar Lipids |
| 1-hexadecanoyl(d31)-2-(9Z-octadecenoyl)-sn-glycero-3-[phospho-L-serine] | 110922, Avanti Polar Lipids |
| 26:0-d4 Lyso PC | 860389, Avanti Polar Lipids |
| 18:1 Chol (D7) ester | 111015, Avanti Polar Lipids |
| cholest-5-en-3ß-ol(d7) | LM-4100, Avanti Polar Lipids |
| D-erythro-sphingosine-d7 | 860657, Avanti Polar Lipids |
| D-erythro-sphingosine-d7-1-phosphate | 860659, Avanti Polar Lipids |
| N-palmitoyl-d31-D-erythro-sphingosine | 868516, Avanti Polar Lipids |
| N-palmitoyl-d31-D-erythro-sphingosylphosphorylcholine | 868584, Avanti Polar Lipids |
| Octadecanoic acid-2,2-d2 | 19905-58-9, Sigma Aldrich |


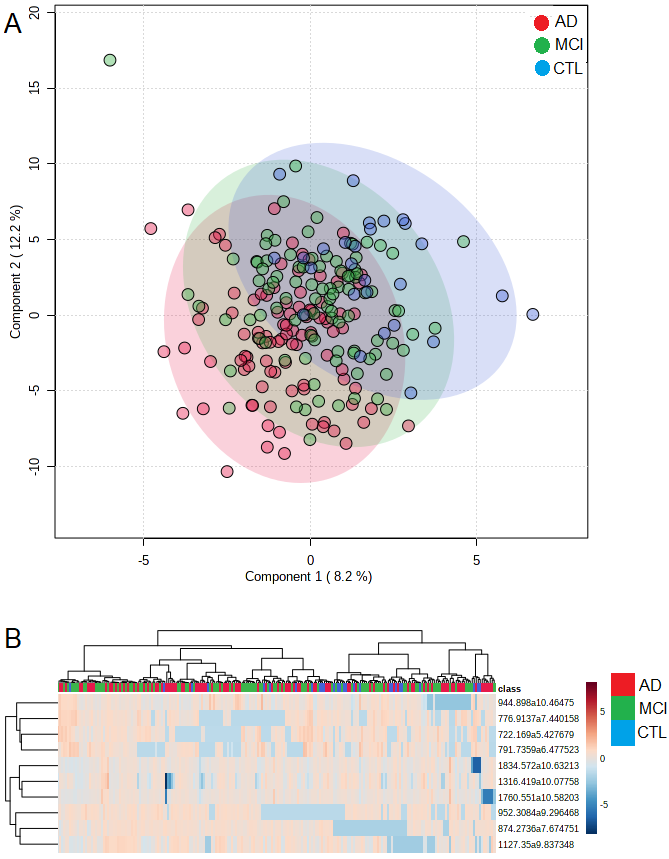


**Supplementary Figure 1.** CSF lipidomic profile of AD, MCI and control (CTL) subjects. (A) Partial least squares-discriminant analysis for the diagnostic groups. (B) Heat map representation of 10 lipids with significantly different (not FDR adjusted) levels between diagnostic groups.


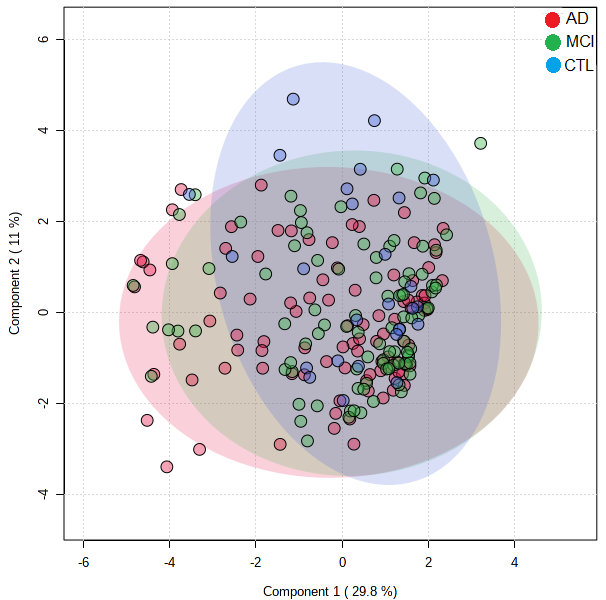


**Supplementary Figure 2.** Partial least squares-discriminant analysis (PLS-DA) of CSF lipidome profile of patients with AD, MCI and control subjects detected in negative ionization mode.

**Supplementary Table 2**. CSF lipids associated with pathological levels of CSF AD biomarkers.

|  | **Name** | **Mass** | **RT** | ***p*** | **OR** | **95% CI for OR** |
| --- | --- | --- | --- | --- | --- | --- |
| **Aβ42** | **Unknown** | 1612.504 | 10.43 | 0.001 | 3.347 | 1.277 – 8.771 |
|  | **Unknown** | 776.9137 | 7.44 | 0.010 | 2.626 | 1.003 – 6.877 |
|  | **Unknown** | 339.3383 | 3.92 | 0.007 | 0.366 | 0.140 – 0.957 |
|  | **Unknown** | 757.2425 | 8.75 | 0.003 | 3.033 | 1.156 – 7.956 |
|  | **Unknown** | 364.3928 | 3.01 | 0.001 | 4.054 | 1.394 – 11.788 |
|  | **Unknown** | 854.2311 | 6.68 | 0.000 | 0.187 | 0.059 – 0.586 |
|  | **Unknown** | 784.3074 | 7.13 | 0.007 | 3.097 | 1.045 – 9.177 |
|  | **Unknown** | 874.2736 | 7.67 | 0.007 | 3.131 | 1.044 – 9.388 |
|  | **Unknown** | 1090.247 | 7.75 | 0.026 | 2.420 | 1.113 – 5.259 |
|  | **PC(O-36:3)/PC(P-36:2)** | 751.5893 | 9.16 | 0.043 | 0.450 | 0.208 – 0.974 |
|  | **Unknown** | 1257.203 | 11.23 | 0.024 | 2.303 | 1.117 – 4.749 |
| **Ptau** | **Unknown** | 1334.428 | 8.58 | 0.005 | 0.078 | 0.008 – 0.809 |
|  | **Unknown** | 500.4377 | 4.43 | 0.007 | 0.029 | 0.001 – 0.835 |
|  | **Unknown** | 776.9137 | 7.44 | 0.003 | 36.069 | 1.658 – 784.822 |
|  | **Unknown** | 452.4514 | 5.65 | 0.010 | 7.127 | 1.006 – 50.499 |
|  | **Unknown** | 1482.468 | 8.93 | 0.005 | 0.127 | 0.019 – 0.834 |
| **Ttau** | **Unknown** | 636.5484 | 8.21 | 0.047 | 2.121 | 1.010 – 4.455 |

Aβ42: amyloid beta 1-42; Ptau: phosphorylated tau; Ttau: total tau; PC(O): ether-linked phosphatidylcholine; PC(P): phosphatidylcholine plasmalogen; RT: retention time; OR: odds ratio; CI: confidence interval

**Supplementary Table 3**. CSF lipids associated with rate of MCI to AD progression.

| **Name** | **Mass** | **RT** | ***p*** | **OR** | **95% CI for OR** |
| --- | --- | --- | --- | --- | --- |
| **Cer(d36:2)** | 609.5135 | 8.21 | < 0.001 | 0.173 | 0.066 – 0.449 |
| **Unknown** | 1265.302 | 8.29 | 0.011 | 2.449 | 1.226 – 4.892 |
| **PA(42:5)** | 760.5294 | 8.73 | 0.023 | 2.227 | 1.119 – 4.430 |
| **PA(46:7)** | 830.605 | 9.17 | 0.017 | 2.542 | 1.184 – 5.455 |
| **Unknown** | 1257.203 | 11.23 | 0.014 | 0.442 | 0.231 – 0.845 |

Cer: ceramide; PA: phosphatidic acid; RT: retention time; OR: odds ratio; CI: confidence interval
